# Response of Patients with Color Vision Defects to Worth 4-Dot and Duochrome Tests

**DOI:** 10.3390/healthcare12222262

**Published:** 2024-11-13

**Authors:** Ali Almustanyir, Balsam Alabdulkader, Muteb Alanazi, Abdulmalik Alhadyani, Meznah S. Almutairi, Mohammed Alhazmi, Essam Almutleb, Tahani Alqahtani, Bader Almagren, Mosaad Alhassan

**Affiliations:** Department of Optometry, College of Applied Medical Sciences, King Saud University, Riyadh 11451, Saudi Arabia; alabdulkader@ksu.edu.sa (B.A.); mkalanazi@ksu.edu.sa (M.A.); 439100115@student.ksu.edu.sa (A.A.); mzalmutairi@ksu.edu.sa (M.S.A.); malhazmyi@ksu.edu.sa (M.A.); esalsarhani@ksu.edu.sa (E.A.); talqahtani@ksu.edu.sa (T.A.); balmagren@ksu.edu.sa (B.A.); malhassan@ksu.edu.sa (M.A.)

**Keywords:** color vision defect, Worth 4 dots, duochrome test, red–green color defect, color vision testing

## Abstract

**Background:** Individuals with congenital color vision defects (CVDs) are at greater risk of misidentifying colors, necessitating an investigation into their ability to distinguish colors accurately. This study aimed to assess how individuals with CVDs perceive the colors in the Worth four-dot (W4D) and duochrome tests. It also explored whether individuals with CVDs require more detailed instructions from optometrists and eye care providers during these tests. **Method**: Thirty-two participants with congenital CVDs were recruited for this study. Participants with ocular diseases were excluded based on a brief questionnaire. The participants underwent a W4D test to determine the colors of four circles and the duochrome test without using filters. **Result:** All the participants correctly identified the W4D colors, except for five participants (15.62%). Furthermore, among all the participants, two (one with deuteranopia and one with protanopia) failed to identify the colors correctly in the duochrome test (6.25%). **Conclusions:** The findings suggest that in the context of optometric assessment, it could be essential for optometrists to incorporate a preliminary inquiry into a patient’s ability to identify colors prior to performing diagnostic tests such as the W4D or duochrome tests. Incorrect responses from patients with CVDs might mislead optometrists regarding the proper outcomes of the test.

## 1. Introduction

Color vision perception is the ability of an organism or individual to distinguish and interpret different colors in its environment. It is a complex process that involves the eyes, the brain, and various optical and neural mechanisms. Understanding color vision is crucial in several fields, such as neuroscience, psychology, and visual media design [1,2,3].

Individuals with difficulty distinguishing color shades are typically described as having color vision deficiency (CVD). The most prevalent type of CVD is red–green CVD, a genetic condition that affects the perception of red and green hues. These deficiencies are diagnosed using color vision tests, which can help determine the type and severity of the deficiency, such as red–green or blue–yellow deficiencies. CVDs can significantly affect daily activities such as driving and can be critical in certain professions [4].

Color vision testing is an essential component of a comprehensive eye examination. It is typically used to assess an individual’s ability to perceive and distinguish colors accurately. The Ishihara is a common color vision test used to detect CVDs. It utilizes a series of plates with numbers or patterns composed of dots in various colors to determine the presence of CVDs. Anomaloscopy, the gold standard for determining the type and severity of red–green CVD, is less commonly available in clinical settings [5].

The Ishihara test is probably the most common color vision test used in clinical settings. It uses numbers to detect CVDs and has multiple colors in the background. Also, the numbers are presented in different colors [6]. The Hardy, Rand, and Rittler (HRR) color vision test is a common test used to diagnose CVDs, either congenital or acquired. This test uses a gray background that has three different symbols (circle, triangle, and cross) with 24 plates. The symbols differ in brightness and colors and are randomized throughout the test. Individuals are asked to identify the symbols correctly to determine whether they have CVDs. This test is also used to assess the severity of the CVD and decide whether or not they have red–green or blue–yellow defects [7].

In optometry clinics, some visual assessments involve red–green targets that require normal color vision perception. For example, the Worth four-dot (W4D) test is used to assess binocular vision functions, such as suppression and double vision, by presenting dissociated views to each eye using red–green targets and red–green filters. The W4D test consists of four circles or shapes: two green circles or crosses, one red or diamond, and one white circle. With red–green filters, the green filter allows only the green and white targets to be seen and the red filter allows only the red and white targets to be seen. An individual with normal binocular vision should see four targets: one red circle or a diamond, two green circles or crosses, and a pinky–yellowish circle. If the suppression of one eye occurs, some targets will be missing; if double vision is present, there will be more than four targets [5,6,7,8]. The W4D test is an established method for assessing central and peripheral fusion [7], with dot separation used for the 3m test and a visual angle of 1.25° to evaluate central fusion [7,9,10]. It was believed that patients with CVDs might not be able to undergo W4D testing, as the used targets are red and green [11,12,13]; however, another study, which utilized red–green filters in the W4D testing, demonstrated that patients with variable degrees of congenital red–green CVD and varying degrees of binocularity could successfully perform the W4D test, showing reliable results consistent with their estimated binocular sensory status [14]. Typically, the W4D test involves the eye care provider explaining the procedures to the patients; thus, their responses are critical for accurate interpretation.

The duochrome test is another red–green target-based routine assessment test used in vision testing to refine the final sphere in subjective refraction, aiming to prevent undercorrection and overcorrection. The test presents the patient with black letters or symbols on a red and green background. It capitalizes on the eye’s longitudinal chromatic aberration, which causes shorter wavelengths (green) to focus in front of the longer red wavelengths (red). Optimal vision is assumed when the letters or symbols appear equally sharp on both the red and green sides [15].

This study aims to evaluate how patients with CVDs perceive and distinguish colors when subjected to the W4D and duochrome tests. The contribution of this study is to investigate whether tests that rely on color perception, such as the W4D and duochrome tests, are suitable for routine use in clinical optometry and eye care or if eye care providers need to implement additional measures to ensure the accuracy and effectiveness of these assessments in clinical settings.

## 2. Methods

### 2.1. Subjects

A total of 32 participants with congenital CVDs were recruited for this cross-sectional study. Participants with ocular diseases were excluded based on a brief questionnaire (Appendix A). An exclusion was also made for all people with normal color perception. The possibility of a bilateral disorder associated with an acquired color vision defect was further reduced by restricting the subject pool to only those with a monocular visual acuity of at least 6/6 in the better eye and 6/9 in the other eye at 6 m with or without spectacles or contact lenses. This study adhered to the principles of the Declaration of Helsinki and was approved by the King Saud University Office of Research Ethics (E-22-6663). All the participants provided written informed consent after the nature and purpose of the study were described.

### 2.2. Data Collection Procedure

**Figure 1:**
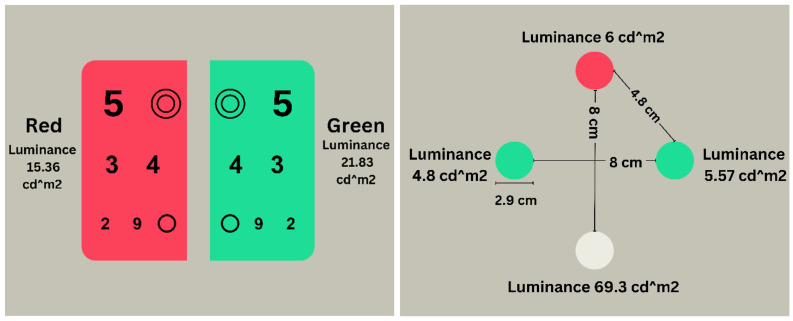
Illustrations of the Worth 4-dot (**left**) and duochrome tests (**right**) used in this study. The third author captured the image.

**Figure 2:**
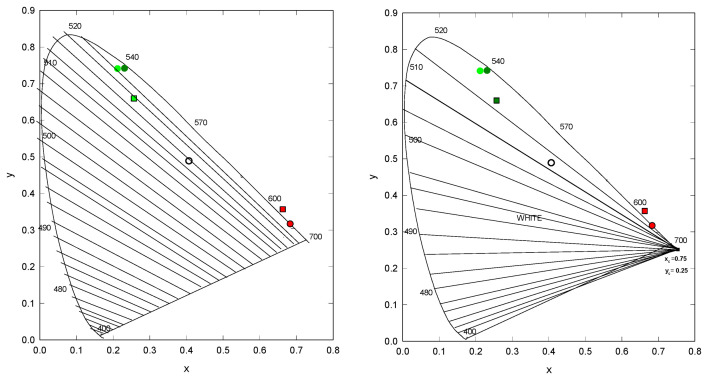
Deutan (**right**) and protan (**left**) lines of confusion. These lines are based on the copunctal points identified by Vos and Walraven, and the spacing between the lines is based on the dichromatic wavelength discrimination data provided by Pitt and Wright [16,17,18]. The chromaticity coordinates for the colors of the circles in the W4D test are red, light green, dark green, and white. The red and green squares represent the chromaticity coordinates for the red and green areas in the duochrome tests.

The W4D test was administered using the luminance shape provided by Topcon autochart projectors Topcon, Tokyo, Japan, model number ACP-8). The test displayed four circles, each with a diameter of 2.9 cm. The configuration included two green circles, one white circle, and one red circle (Figure 1). All circles were equidistant from each other, spaced 8 cm apart both horizontally and vertically. Participants were tasked to determine the color of the four circles without the use of filters. The room light was dim (1 lux). This step was performed to ascertain how participants detected these colors. Each participant was asked to name the color of each circle. During this identification process, participants were not informed of the potential color choices being red, green, or white. The duochrome test, obtained from the Topcon projector used for the W4D test in this study, features black circles and numbers on a red background on the right side and a green background on the left side (Figure 2).

One of the most common methods for determining whether colors are confused by participants with CVDs is to plot the chromaticity coordinates of any color on the lines of confusion in a CIE diagram. If the colors fall along the same line of confusion and have the same brightness, they appear identical. However, the greater the distance between the two colors, the less likely it is that individuals with milder defects will confuse them. The straight lines in Figure 2 represent the color confusion lines for those with protanopia and deuteranopia [16,17,18]. The chromaticity coordinates of the W4D and duochrome test colors are shown in Figure 2. Only the red and left-hand green circles were on the same line of confusion for protanopia and deuteranopia.

#### 2.2.1. Color Tests

CVDs were classified according to Rayleigh color matching using the HMC Oculus anomaloscope (Oculus Optikgeräte GmbH, Wetzlar, Germany). Tinted contact lenses and spectacles were not utilized. In addition, pseudoisochromatic plate tests were employed to classify CVDs.

#### 2.2.2. Pseudoisochromatic Tests

The 38-plate edition of the Ishihara test (Kanhara, & Co., Ltd., 1996, Tokyo, Japan) and the Hardy, Rand, and Rittler test (4th edition, Richmond Products, Albuquerque, NM, USA) were used. Participants were instructed to read the numbers through the Ishihara pages and detect the symbols in the HRR test, and their responses were recorded on a record sheet. The viewing distance was approximately 50 cm for all tests. The sequence of all the tests was randomized using a random block design. The failure criterion for the Ishihara screening plates was ≥3 errors on plates 1–17, >2 errors on the red–green screening plate, and >0 errors on the blue–yellow HRR plates. The illuminance for the tests was 1000 lx (±5%) in the horizontal plane.

#### 2.2.3. Farnsworth D15 (D15)

The instructions were based on the recommendations by Dain et al. (2019) [19]. Fifteen loose caps were randomly arranged on a table in front of the participant. The participants were then instructed to organize these loose caps according to color similarity, starting with the fixed reference cap in the box. Rearrangement of the caps was allowed. The test is usually analyzed by counting the number of crossings and transpositions. A major crossing is defined as a difference between adjacent cap numbers that is greater than two. The transposition occurs when a cap, or rectangle, is placed in only one to two positions from the correct arrangement. Commonly, more than one major crossing is considered a failure [19].

## 3. Results

A total of 32 individuals with CVDs participated in this study, with a mean age of 21.56 years (±6.01). The participants’ demographic data are summarized in Table 1. Based on the anomaloscopic results, there were 6 participants with deuteranopia (18.75%), 13 who were deuteranomalous (40.62%), 4 with protanopia (12.5%), and 9 who were protanomalous (28.13%).

All the participants correctly identified the W4D colors, except for five (two with protanopia, one with deuteranopia, and one who was protanomalous) with a percentage of 15.62%. Furthermore, of all the participants, two (one with deuteranopia and one with protanopia) failed to correctly identify the colors in the duochrome test, with a percentage of 6.25%. These exceptions were not significantly different. The responses of the five participants to the W4D and duochrome test colors are presented in Table 1. The majority of those in the protanopia group (75%) had an error in either the W4D or the duochrome test. Furthermore, Table 1 demonstrated that the anomaloscope’s range is at the maximum for four cases in which an error was made in either the W4D or the duochrome test.

The white circle in the W4D test was the target that the participants could not identify correctly; three made errors, and two of them made the same mistake. The red side of the duochrome test was the easiest for them to identify. None of the study participants misidentified the color.

## 4. Discussion

### 4.1. Evaluation of the Accomplished Work

The literature has shown limited studies on conducting the W4D and duochrome tests in patients with red–green CVDs. When performing the W4D tests, the right eye is covered with a red filter and the left eye with a green filter. It may be considered that CVDs could create difficulties in the W4D test, as the colors used in the test are red and green [20,21].

The results of this study revealed that slightly more than 15% of the individuals with CVDs incorrectly identified the colors in the W4D and duochrome tests, with brown being the most commonly reported error. It seems that the errors were not based on random guesses by these participants but rather on their perception of the color brown. These participants expressed surprise when they were informed of the correct color.

The identification of colors by individuals with CVDs appears idiosyncratic and context dependent [15,22,23]. In this study, most participants correctly named the colors in the W4D and duochrome tests; however, an exception was noted in four participants with dichromacy and one with anomalous trichromacy, which indicated that their incorrect responses were related to their experience of seeing similar colors before, including pink, brown, and light brown. However, it is noteworthy that these participants were taking these tests for the first time.

The chromaticity coordinates of the W4D colors and duochrome tests were not on the same lines of confusion for protanopia and deuteranopia, with exceptions being the red and green in the right circle of the W4D test and the green color in the duochrome test. Three participants with CVDs identified red as pink in the W4D test. In addition, only one participant correctly identified the left-hand green circle as brown but called it a red circle. Although the red and left-hand green circles were on the same line of confusion and were far apart, they differed in luminance. The researchers did not inquire why participants believed the color was brown. The failure of participants to name the color correctly may have been due to the presence of another green color. It is speculated that the participants may have thought that these colors looked different and should not be named green. Our results further demonstrate that individuals with CVDs make errors in identifying signal colors that fall near the dichromatic lines of confusion [24,25,26]. Supposing the CVD population uses a signaling system in which the confusion of the signal lights is possible, there should be redundant coding that is obvious or the information conveyed by the confused lights should be similar. Finally, asking an individual with a CVD to identify the color may allow clinicians to explain which colors their patient will likely confuse. Even if the patient correctly identifies the test colors, the clinician could explain why the question was asked as part of their counseling.

### 4.2. Limitations

One of the limitations of this study is that color filters were not used in the W4D test, which typically assesses binocular vision functions by dissociating viewing between the two eyes using red–green targets and red–green filters. Participants with CVDs were tested without the filters to focus specifically on determining their chromatic responses. Although the traditional use of filters in the W4D test serves to evaluate binocular vision by ensuring each eye only sees certain colors, the exclusion of filters in this study was intentional and aligned with our research objectives. The primary goal was to examine how individuals with CVDs perceive colors, rather than to assess their binocular vision capabilities. Therefore, the absence of filters does not detract from the validity of our findings regarding color perception, as this was the central aspect under investigation. Consequently, it remains unclear whether incorrectly identifying the colors in these tests negatively affects the clinical results obtained from these results. Future studies are warranted to investigate whether results differ among individuals with CVDs when using such filters. Other limitations are the small sample size and the exclusion of individuals with acquired red–green CVDs. This includes severe types of CVDs such as achromatopsia and other diseases accompanying CVDs such as cone dystrophy or cerebral achromatopsia. Additionally, there were no patients with strabismus or amblyopia. Further studies involving a larger number of patients are necessary to determine whether patients with variable red–green color vision defects and abnormal binocular sensory status resulting from strabismus or amblyopia can yield predictable results with the W4D test. More studies can be performed using filters in the W4D test to investigate any relationship between the binocular status and CVDs.

### 4.3. Sensitivity Analysis

Red–green CVDs are the most common types, and severe cases can affect the patient in daily life activities. In an optometry clinic, a few of the assessments use colors, and false positive outcomes must be eliminated. Furthermore, these clinical tests depend on correctly identifying the colors red and green [17,24]. Asking a patient with CVDs, especially those with advanced red–green deficiency, to identify the colors in the W4D or duochrome tests may provide clinicians with an opportunity to explain which colors their patient are likely to confuse and then perform diagnostic color vision tests. Even if the patient correctly identifies the colors, the clinician could explain why the question was asked as part of their counseling.

The findings of this study confirmed that the majority of individuals with color vision defects (CVDs) were able to accurately identify colors in the W4D test, suggesting that individuals with CVDs are generally capable of performing color-dependent tasks such as the duochrome test, which only two subjects from the entire study group failed to perform correctly. These results indicate that individuals with CVDs can still perceive colors sufficiently to participate in standard clinical tests. Therefore, if a patient with undiagnosed CVDs were to undergo a duochrome test as part of subjective refraction in a clinical setting, it is reasonable to conclude that the results of the duochrome test would be reliable. In the context of optometric assessment, it could be essential for optometrists to incorporate a preliminary inquiry into a patient’s ability to identify colors prior to performing diagnostic tests such as the W4D or duochrome tests. Incorrect responses from patients with CVDs might mislead optometrists regarding the proper outcomes of the test. This insight is crucial for optometrists, as it underscores the ability of patients with CVDs to engage in routine optometric assessments without significantly compromising the accuracy of the test outcomes. This finding supports the inclusion of such tests in standard optometric practice, even for patients with undetected CVDs.

## 5. Conclusions

The findings of this study showed that most of the participants with CVDs could accurately identify the colors in the W4D and duochrome tests, with some exceptions. This suggests that individuals with CVDs can correctly perform color-dependent tasks such as the duochrome test. However, optometrists should treat CVDs with extra care in their assessment and use color-dependent tasks for optimum outcomes. For example, in the duochrome test, optometrists can change their instructions for individuals with CVDs from saying “Which color is better, red or green?” to saying “Which side is better, the right or the left?”, giving the participants more flexibility for their answers. Future research could include the use filters in the W4D test, with a larger sample size including all the types of CVDs.

## Figures and Tables

**Table 1:** The responses of the five participants with CVDs who incorrectly named the colors on the W4D and duochrome tests.

	Case 1	Case 2	Case 3	Case 4	Case 5
Age (years)	20	21	25	14	19
Diagnosis	Protanomaly	Protanopia	Protanopia	Deuteranopia	Protanopia
Anomaloscope results	Lower: 34 Upper: 49 Midpoint: 45	Lower: 0 Upper: 73 Midpoint: NA *	Lower: 0 Upper: 73 Midpoint: NA	Lower: 0 Upper: 73 Midpoint: NA	Lower: 0 Upper: 73 Midpoint: NA
W4D upper red circle	Pink	Red	Red	Red	Red
W4D lower white circle	Light brown	Pink	Pink	White	White
W4D right green circle	Green	Green	Green	Brown	Green
W4D left green circle	Green	Green	Green	Brown	Green
Duochrome (green)	Green	Green	Green	Brown	Light brown
Duochrome (red)	Red	Red	Red	Red	Red
Ishihara	4 errors	16 errors	16 errors	16 errors	15 errors
HRR	3 errors	3 errors	5 errors	6 errors	6 errors
D15	1 crossing 5 transpositions	9 crossings 1 transposition	12 crossings	10 crossings	8 crossings 1 transposition

* NA means that the participant with CVD accepted all the possible mixtures of red and green.

## Data Availability

No new data were created or analyzed in this study. Data sharing is not applicable to this article.

## Appendix A

**Table:**

Questionnaire for Participants with CVDs			
	Question	Yes	No
1	Are you being treated for or do you have glaucoma, optic neuritis, multiple sclerosis, or diabetes?		
2	Other than wearing spectacles or contact lenses and/or having a color vision problem, are you aware of any other vision problems?		
4	Do you have any one of these conditions? (circle): lupus, malaria, rheumatism, and/or any heart condition.		
5	Have you had cataract surgery?		
6	Do you use any of these medications? (circle): chloroquine, hydroxychloroquine “Plaquenil” or digitalis “Digoxin”		
